# The *Anemonia sulcata* Toxin BDS-I Protects Astrocytes Exposed to Aβ_1–42_ Oligomers by Restoring [Ca^2+^]_i_ Transients and ER Ca^2+^ Signaling

**DOI:** 10.3390/toxins13010020

**Published:** 2020-12-31

**Authors:** Ilaria Piccialli, Valentina Tedeschi, Francesca Boscia, Roselia Ciccone, Antonella Casamassa, Valeria de Rosa, Paolo Grieco, Agnese Secondo, Anna Pannaccione

**Affiliations:** 1 Division of Pharmacology, Department of Neuroscience, Reproductive and Dentistry Sciences, School of Medicine, Federico II University of Naples, 80131 Napoli, Italy; piccialli.ilaria@gmail.com (I.P.); valentina.tedeschi@unina.it (V.T.); boscia@unina.it (F.B.); cicconeroselia@gmail.com (R.C.); antonella.casamassa@unina.it (A.C.); biovalder@libero.it (V.d.R.); 2 Department of Pharmacy, School of Medicine, Federico II Universityof Naples, 80131 Napoli, Italy; paolo.grieco@unina.it

**Keywords:** astrocytes, Aβ_1–42_ oligomers, BDS-I, [Ca^2+^]_i_ transients, ER stress, K_V_3.4 channel

## Abstract

Intracellular calcium concentration ([Ca^2+^]_i_) transients in astrocytes represent a highly plastic signaling pathway underlying the communication between neurons and glial cells. However, how this important phenomenon may be compromised in Alzheimer’s disease (AD) remains unexplored. Moreover, the involvement of several K^+^ channels, including K_V_3.4 underlying the fast-inactivating currents, has been demonstrated in several AD models. Here, the effect of K_V_3.4 modulation by the marine toxin blood depressing substance-I (BDS-I) extracted from *Anemonia sulcata* has been studied on [Ca^2+^]_i_ transients in rat primary cortical astrocytes exposed to Aβ_1–42_ oligomers. We showed that: (1) primary cortical astrocytes expressing K_V_3.4 channels displayed [Ca^2+^]_i_ transients depending on the occurrence of membrane potential spikes, (2) BDS-I restored, in a dose-dependent way, [Ca^2+^]_i_ transients in astrocytes exposed to Aβ_1–42_ oligomers (5 µM/48 h) by inhibiting hyperfunctional K_V_3.4 channels, (3) BDS-I counteracted Ca^2+^ overload into the endoplasmic reticulum (ER) induced by Aβ_1–42_ oligomers, (4) BDS-I prevented the expression of the ER stress markers including active caspase 12 and GRP78/BiP in astrocytes treated with Aβ_1–42_ oligomers, and (5) BDS-I prevented Aβ_1–42_-induced reactive oxygen species (ROS) production and cell suffering measured as mitochondrial activity and lactate dehydrogenase (LDH) release. Collectively, we proposed that the marine toxin BDS-I, by inhibiting the hyperfunctional K_V_3.4 channels and restoring [Ca^2+^]_i_ oscillation frequency, prevented Aβ_1–42_-induced ER stress and cell suffering in astrocytes.

## 1. Introduction

Astrocytes, the most abundant glial cells in the central nervous system (CNS), may support neuronal homeostasis, not only releasing trophic factors, but also regulating neurotransmission and synaptic plasticity [1,2,3,4,5,6,7]. Moreover, astrocytes continuously handle gliotransmitters like purines, D-serine, and glutamate, whose uptake and release are regulated by the frequency of their intracellular calcium concentration ([Ca^2+^]_i_) transients, a complex ionic phenomenon involving both extracellular and intracellular compartments including the endoplasmic reticulum (ER).

[Ca^2+^]_i_ transients in astrocytes spread throughout gap junctions belonging to the astrocytic syncytium [8,9,10]. Clearly, the extent of this electric phenomenon is driven by several molecular mechanisms residing in astrocytes [11,12,13]. Firstly, [Ca^2+^]_i_ transients may be generated by the release of Ca^2+^ from ER [14] or by an influx from the extracellular space through ionotropic receptors or plasma membrane channels [12]. While the origin of ER-mediated Ca^2+^ transients seems to be predominant in the soma, the plasmalemmal-located entry mechanisms are also involved at the level of astrocyte processes [13,15,16,17,18].

Since 1965, it has been established that astrocytes can modulate neuronal activity by buffering plateau level of extracellular K^+^ in the brain, thus controlling neuronal excitability [19,20]. Accordingly, defects in astrocytic K^+^ buffering properties have been linked to epilepsy [21] and ischemia-dependent cell death [22]. Although well-established at neuronal level, the involvement of voltage-gated potassium (K_V_) channels in the regulation of the action potential frequency and [Ca^2+^]_i_ transients in astrocytes is far from being fully understood, especially during the neurodegenerative process.

Moreover, defects in membrane excitability and calcium signaling represent fundamental features of many neurodegenerative disorders, including Alzheimer’s disease (AD) [23]. However, how this may include the Ca^2+^ signaling machinery in astrocytes is quite obscure. In this respect, disturbance of ER Ca^2+^ homeostasis determines β-amyloid (Aβ) oligomer-dependent upregulation of glial fibrillary acidic protein (GFAP) expression, a widely recognized marker of astrocytic defects [24].

During neurological diseases, astrocytes undergo complex changes, which are sub-classified into: (1) reactive astrogliosis, an evolutionarily conserved defensive rearrangement of cellular phenotype aimed at neuroprotection, (2) pathological remodeling, when astrocytes acquire new features driving pathology, and (3) astrodegeneration, a type of astroglial atrophy characterized by a loss of homeostatic functions [25].

In particular, astrocyte dysfunction emerges early in AD, and may contribute to its severity and progression [26]. Indeed, prominent astroglia degeneration contributes to neural deficits and cognitive decline [27] through inflammatory response activation and Aβ plaque evolution [28,29].

Initial glia dysfunction, characterized by activated astrocytes expressing elevated levels of GFAP, appears in the very early stages of AD [30]. Interestingly, under these conditions, both genetic deletion and pharmacological inhibition of K_V_3.4 channel subunits, by possibly counteracting cytoplasmic K^+^ loss, are effective in reducing astrocytes’ activation [30]. Interestingly, low cytoplasmic K^+^ concentrations determine the activation of inflammasome [31,32], mainly involved in AD progression [33,34].

Furthermore, K_V_3.4 channels mediating fast inactivating K^+^ currents (I_A_)—mainly contributing to the action potential repolarization—are dysfunctional in AD [30,35,36,37]. Moreover, cell death caused by the loss of cytoplasmic K^+^ concentrations due to the enhanced function of K_V_3.4 channel upon Aβ_1–42_ exposure has been detected at the neuronal level [37]. Accordingly, a significant correlation has been ascertained between altered intracellular K^+^ concentrations and apoptotic processes [38]. In fact, in hippocampal neurons, K_V_3.4 channel blockade prevents the apoptotic cascade triggered by Aβ_1−42_ [37]. Therefore, K_V_3.4 channel could be considered a new pharmacological targetagainst AD progression.

In this respect, toxins from marine organisms with a high selectivity towards ion channels [37,39,40] may provide molecular tools to treat ion channel-related diseases [41].

Therefore, in the present study, we have investigated the effect of the *Anemonia sulcata* toxin blood depressing substance-I (BDS-I) blocking K_V_3.4 channel subunits on [Ca^2+^]_i_ transients, ER Ca^2+^ signaling, reactive oxygen species (ROS) production, and cell survival in cortical astrocytes exposed to Aβ_1–42_ oligomers.

## 2. Results

### 2.1. Exposure to Aβ_1–42_ Oligomers Upregulated K_V_3.4 Protein Expression and Activity in Activated Rat Primary Cortical Astrocytes

**Figure 1:**
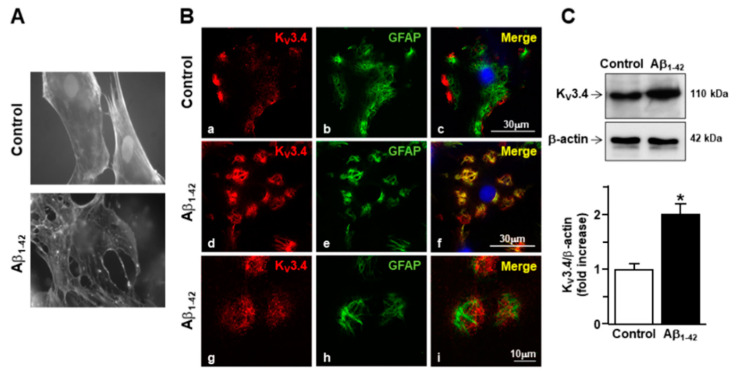
Effect of Aβ_1–42_ oligomers on the upregulation of K_V_3.4 protein expression in rat primary astrocytes. (**A**) Phalloidin-stained images of rat primary astrocytes under control conditions and 48 h after 5 µM Aβ_1–42_ exposure. Nuclei were marked by Hoechst 33258. (**B**) Confocal double immunofluorescence images of rat primary astrocytes displaying both K_V_3.4 (red) and glial fibrillary acid protein (GFAP) (green) immunoreactivities under control conditions (**a**–**c**) or 48 h after 5 µM Aβ_1–42_ exposure (**d**–**i**). Higher magnification of the frame depicted in **g**–**i** displaying intense immuno-positive fiber bundles double-labeled for both K_V_3.4 and GFAP in AD astrocytes. Scale bars: **a**–**f**: 30 µm; **g**–**i**: 10 µm. (**C**) Western blotting and densitometric analysis of K_V_3.4 protein levels under control conditions or 48 h after 5 µM Aβ_1–42_ exposure. Data were normalized on the basis of β-actin and expressed as fold increase compared to control’s expression of three different preparations. * *p* < 0.05 vs. control.

**Figure 2:**
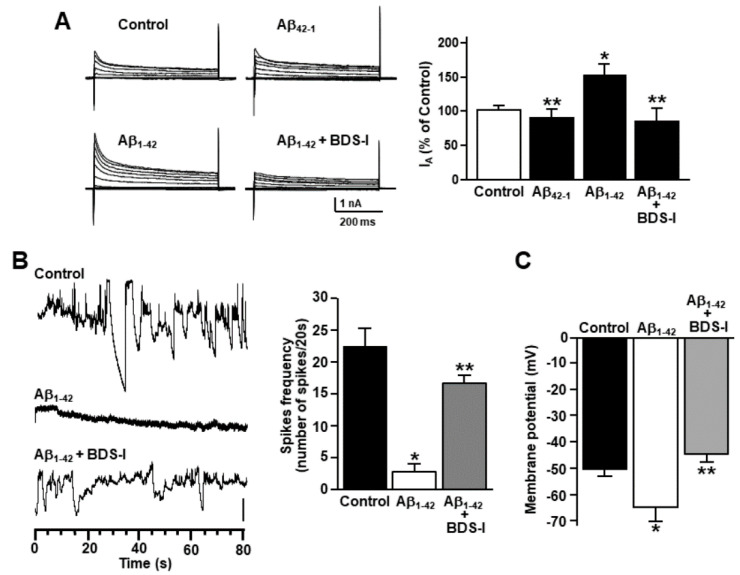
Effect of Aβ_1–42_ oligomers on the upregulation of K_V_3.4 channel activity in rat primary astrocytes. (**A**) Representative traces and quantification of K_V_3.4-mediated fast inactivating K^+^ currents (I_A_) recorded from rat primary astrocytes under control conditions, 48 h after 5 µM Aβ_1–42_ exposure in the absence and in the presence of 50 nM blood depressing substance-I (BDS-I), or 48 h after 5 µM Aβ_42–1_ exposure. The peak values of I_A_, measured at the beginning of the +40mV depolarizing pulse, are expressed as percentage mean ± standard error of the mean (SEM) of three independent experiments performed on three different preparations (*n* = 12 cells in each cell culture and for each group). * *p* < 0.05 vs. controls; ** *p* < 0.05 vs. Aβ_1–42_. (**B**) Representative current traces recorded in the gap-free mode under control conditions or 48 h after 5 µM Aβ_1–42_ exposure in the absence and in the presence of 50 nM BDS-I in rat primary astrocytes. Vertical scale bar below the trace represents 1 pA. (**C**) Quantification of the effects of Aβ_1–42_ oligomers on membrane potential under control conditions or 48 h after 5 µM Aβ_1–42_ exposure in the absence and in the presence of 50 nM BDS-I. The values are expressed as mV and represent the mean ± SEM of three independent experiments performed on three different preparations (for both **B** and **C**: *n* = 12 cells for each group). * *p* < 0.05 vs. controls; ** *p* < 0.05 vs. Aβ_1–42_.

To examine cytoskeleton rearrangement after exposure to Aβ_1–42_ oligomers, F-actin cytoskeleton was stained in primary cortical astrocytes with the actin-binding dye phalloidin. The filament network was brightly stained in control astrocytes (Figure 1A). However, actin bundle disassembly and body extroflections revealed pronounced astrogliosis in astrocytes treated with 5 µM Aβ_1–42_ oligomers for 48 h (Figure 1A). Confocal double immunofluorescence experiments revealed that the K_V_3.4 immunoreactivity was mainly confined along the plasma membrane of control GFAP-positive cortical astrocytes (Figure 1B). Moreover, primary cortical astrocytes exposed to 5 µM Aβ_1–42_ oligomers for 48 h showed a more pronounced immunoreactivity for both GFAP and K_V_3.4 as compared to controls (Figure 1B). Interestingly, the two immunosignals intensely overlapped within the filament bundles (Figure 1B). Furthermore, Western blotting analysis revealed that after 48 h exposure to Aβ_1–42_ oligomers, K_V_3.4 protein expression significantly increased in primary cortical astrocytes as compared to controls (Figure 1C). Accordingly, patch-clamp recordings in whole-cell configuration showed a significant upregulation of the fast-inactivating currents (I_A_) in primary cortical astrocytes exposed for 48 h to Aβ_1–42_ oligomers compared with control astrocytes (Figure 2A). In contrast, 5 μM of the scramble Aβ peptide (Aβ_42–1_) did not induce any significant modification of I_A_ (Figure 2A). Moreover, the contribution of K_V_3.4 to I_A_ upregulation in primary cortical astrocytes exposed to Aβ_1–42_ oligomers was further tested by blocking this channel with the selective blocker BDS-I. Treatment with BDS-I (50 nM) reduced I_A_ (Figure 2A) in primary cortical astrocytes exposed to Aβ_1–42_ oligomers (Figure 2A). Interestingly, electrophysiological patch-clamp recordings in primary cortical astrocytes exposed to Aβ_1–42_ oligomers (5 μM, 48 h) revealed that firing frequency was lower than in controls (Figure 2B), whereas resting membrane potential was more negative than in controls (Figure 2C). Moreover, BDS-I treatment counteracted either the spike frequency decrease or the membrane hyperpolarization in primary cortical astrocytes exposed to Aβ_1–42_ oligomers (Figure 2B,C, respectively).

### 2.2. Effects of BDS-I on [Ca^2+^]_i_ Transients and ER Ca^2+^Signaling in Rat Primary Astrocytes Exposed to Aβ_1–42_ Oligomers

**Figure 3:**
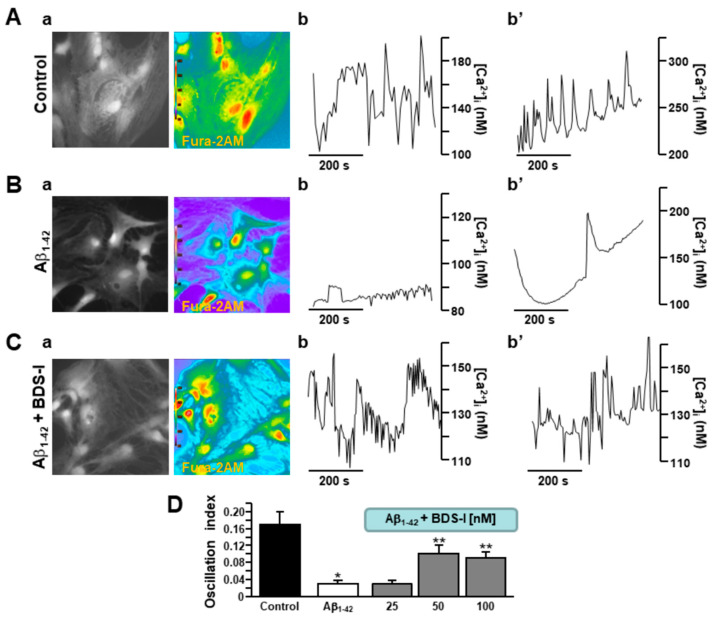
Effects of BDS-I on Aβ_1–42_-dependent modulation of [Ca^2+^]_i_ transients in rat primary astrocytes.(**A**) Representative fluorescent images (**a**) and traces (**b**,**b′**) of rat primary cortical astrocytes in control conditions displaying two different types of [Ca^2+^]_i_ transients. Under control conditions, *n* = 30 cells have been recorded in three different experimental sessions. (**B**) Representative fluorescent images (**a**) and traces (**b**,**b′**) of rat primary cortical astrocytes exposed to Aβ_1–42_ oligomers (5 µM, 48 h) displaying two types of reduced [Ca^2+^]_i_ transients. Under these conditions, *n* = 33 cells have been recorded in three different experimental sessions. (**C**) Representative fluorescent images (**a**) and traces (**b**,**b′**) of rat primary cortical astrocytes exposed simultaneously to Aβ_1–42_ oligomers (5 µM, 48 h) and BDS-I (50 nM) displaying restored [Ca^2+^]_i_ transients. Under these conditions, *n* = 30 cells have been recorded in three different experimental sessions. (**D**) Quantification of the oscillatory index in **A**, **B**, and **C**. The experiments with BDS-I have been performed in the nanomolar range of concentrations. Data are reported as mean ± SEM. * *p* < 0.05 vs. control; ** *p* < 0.05 vs. all.

Rat primary cortical astrocytes displayed spontaneous [Ca^2+^]_i_ transients in the period of control recordings characterized by two main oscillatory patterns with different degrees of irregularity (Figure 3(Aa,b,b′)). After 48 h exposure to Aβ_1–42_ oligomers, the frequency of astrocytic [Ca^2+^]_i_ transients was significantly reduced (Figure 3(Ba,b,b′,D)). In accordance with the electrophysiological experiments aimed to detect spike frequency and membrane potential, BDS-I counteracted the effect of the prolonged exposure to Aβ_1–42_ oligomers on [Ca^2+^]_i_ transients, thus quite restoring the resting frequency in a dose-dependent way (Figure 3(Ca,b,b′,D).

**Figure 4:**
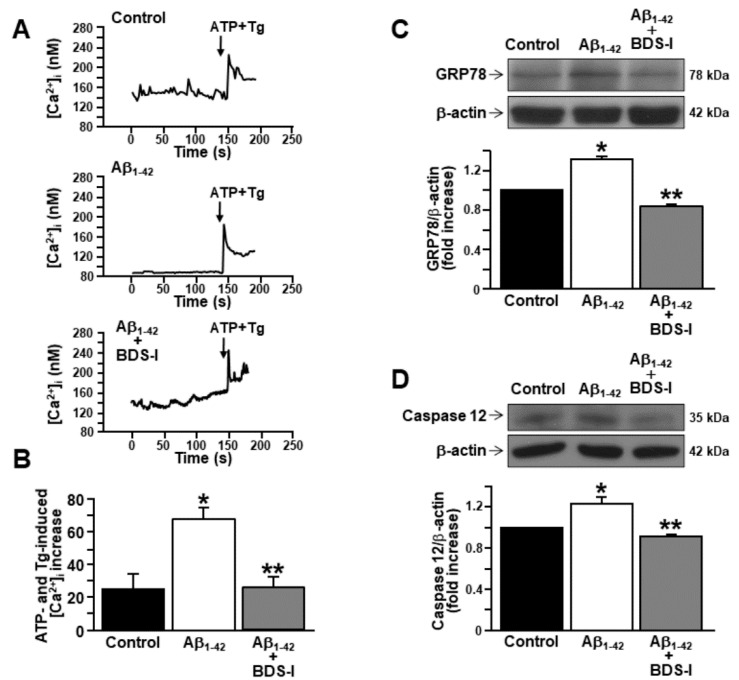
Effects of BDS-I on endoplasmic reticulum (ER) Ca^2+^ level and ER stress markers in rat primary astrocytes exposed to Aβ_1–42_ oligomers.(**A**) Representative traces of the effect of ATP (100 µM) + thapsigargin (Tg, 1 µM) in a nominal Ca^2+^-free solution on [Ca^2+^]_i_ in astrocytes under control conditions (top trace *n* = 40), exposed to Aβ_1–42_ oligomers(5 µM, 48 h) (middle trace, *n* = 48) or exposed to Aβ_1–42_ oligomers (5 µM, 48 h) plus BDS-I (50 nM) (bottom trace, *n* = 50). (**B**) Quantification of A reported as mean±SEM. * *p* < 0.05 vscontrol; ** *p* < 0.05 vs. Aβ_1–42_. (**C**) Representative Western blotting and densitometric quantification of GRP78/BiP protein expression levels under control conditions or 48 h after 5 µM Aβ_1–42_ oligomers exposure in the absence and in presence of 50 nM BDS-I.The values were normalized on the basis of β-actin and expressed as fold increase compared to controls of three independent experimental sessions. * *p* < 0.05 vscontrols; ** *p* < 0.05 vs. Aβ_1–42_. (**D**) Representative Western blotting and densitometric quantification of active caspase 12 under control conditions or 48 h after 5 µM Aβ_1–42_ oligomers exposure in the absence and in presence of 50 nM BDS-I. The values were normalized on the basis of β-actin and expressed as fold increase compared to controls of three independent experimental sessions. * *p* < 0.05 vs. control; ** *p* < 0.05 vs. Aβ_1–42_.

In order to assess the involvement of the major intracellular Ca^2+^-storing organelle in the modulation of [Ca^2+^]_i_ transients by Aβ_1–42_ oligomers, Ca^2+^ depletion from the ER was induced in cortical astrocytes by ATP and thapsigargin in a nominal Ca^2+^-free solution. Interestingly, after 48 h exposure to Aβ_1–42_ oligomers, ER Ca^2+^ content was higher in AD astrocytes than in controls (Figure 4A,B). Moreover, when astrocytes were incubated with BDS-I together with Aβ_1–42_ oligomers, ER Ca^2+^ content was restored to control level (Figure 4A,B), thus suggesting a putative, and possibly compensatory, involvement of the increased K_V_3.4-mediated outward K^+^ currents in the ER Ca^2+^ overload.

### 2.3. Effects of BDS-I on ER Stress Markers in Rat Primary Astrocytes Exposed to Aβ_1–42_ Oligomers

Current evidence suggests that Ca^2+^ dysregulation and ER stress still represent two relevant features of Aβ accumulation [24,42,43]. Therefore, the expression of the ER chaperone GRP78/BiP has been studied in astrocytes exposed to Aβ_1–42_ oligomers (5 μM, 48 h), considering this chaperone as one of the main markers of early AD stage [43]. After 48 h exposure to Aβ_1–42_ oligomers, GRP78/BiP protein expression peaked in AD astrocytes compared with controls (Figure 4C). Importantly, BDS-I prevented GRP78/BiP overexpression when incubated with Aβ_1–42_ oligomers (Figure 4C). Moreover, the expression of active caspase 12 was also upregulated in astrocytes by a long exposure to Aβ_1–42_ oligomers (5 μM, 48 h) (Figure 4D), while it was prevented in astrocytes simultaneously exposed to BDS-I and Aβ_1–42_ oligomers (Figure 4D).

### 2.4. Effects of BDS-I on ROS Production, Mitochondrial Activity, and Lactate Dehydrogenase (LDH) Release in Rat Primary Astrocytes Exposed to Aβ_1–42_ Oligomers

**Figure 5:**
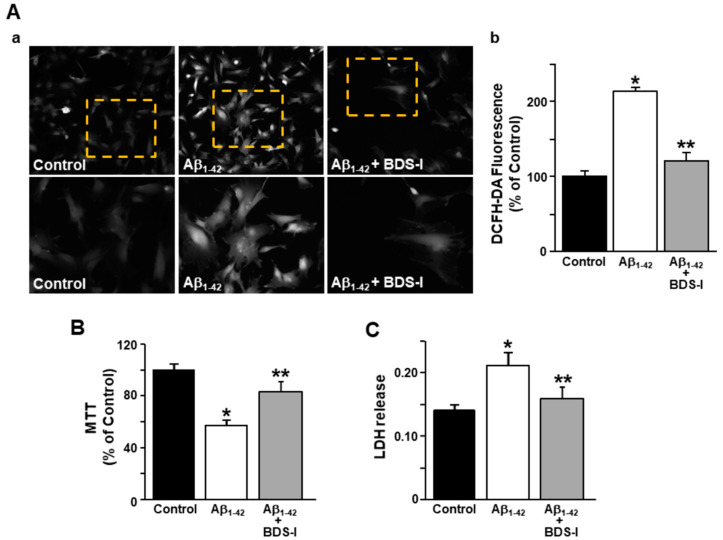
Effects of BDS-I on ROS production, mitochondrial activity, and lactate dehydrogenase (LDH) release in rat primary astrocytes exposed to Aβ_1–42_ oligomers. (**A**) Representative images (**a**) and quantification (**b**) of the fluorescence intensity of 2′,7′-dichlorodihydrofluorescein diacetate (DCFH-DA) under control conditions or 48 h after 5 µM Aβ_1–42_ exposure in the absence and in the presence of 50 nM BDS-I. The values are expressed as percentage mean±SEM of three independent experimental sessions. * *p* < 0.05 vs. controls; ** *p* < 0.05 vs. Aβ_1–42_. (**B**) Quantification of mitochondrial dehydrogenase activity under control conditions or 48 h after 5 µM Aβ_1–42_ exposure in the absence and in the presence of 50 nM BDS-I. The values are expressed as percentage mean ± SEM of three independent experimental sessions. * *p* < 0.05 vs. controls; ** *p* < 0.05 vs. Aβ_1–42_. (**C**) Quantification of LDH release under control conditions or 48 h after 5 µM Aβ_1–42_ exposure in the absence and in the presence of 50 nM BDS-I. The values are expressed as mean ± SEM of three independent experimental sessions. * *p* < 0.05 vs. control; ** *p* < 0.05 vs. Aβ_1–42_.

Considering that ER stress and dysregulated calcium homeostasis may lead to reactive oxygen species (ROS) formation [42], they were detected in rat primary astrocytes using the specific fluorescent dye 2′,7′-dichlorodihydrofluorescein diacetate (DCFH-DA). Furthermore, dysfunction in lactate dehydrogenase (LDH) release and mitochondrial activity were detected as markers of cell suffering in AD astrocytes. ROS production was significantly increased after exposure to Aβ_1–42_ oligomers (5 μM, 48 h) compared with control cells (Figure 5A), while BDS-I prevented this event in AD astrocytes (Figure 5A). Consistently, BDS-I counteracted the significant reduction in mitochondrial activity and the increased level of LDH release induced by Aβ_1–42_ oligomers in astrocytes (Figure 5B,C, respectively).

## 3. Discussion

The present study shows that the *Anemonia sulcata* toxin BDS-I restored spontaneous [Ca^2+^]_i_ transients in rat primary astrocytes exposed to Aβ_1–42_ oligomers. This may occur via the inhibition of the hyperfunctional K_V_3.4 potassium channel, whose expression and activity resulted to be significantly upregulated in Aβ_1–42_-treated astrocytes. Accordingly, the expression and function of K_V_3.4 channel subunits have been previously found to be upregulated in reactive astrocytes in AD Tg2576 mouse brains [30]. The chosen time-point of BDS-I incubation comes from the previous study [30] showing that, nevertheless the upregulation of both K_V_3.4 potassium channel expression and activity starts before, the GFAP-monitored astrogliosis occurs only at 48 h exposure to Aβ_1–42_ oligomers [30].

In line with our results, both astrocytes associated with senile plaques in APP/PS1 mice [44] and astrocytes from APP_Swe_ mice in an early stage of the disease display atypical Ca^2+^ waves [45].

Moreover, the mechanisms of spontaneous astrocytic [Ca^2+^]_i_ transients, mainly involved in important brain functions, are still unclear. In this study, we have identified: (i) a novel mechanism controlling the electrical phenomenon represented by K_V_3.4 channel subunits and (ii) a new putative molecule with a therapeutic profile.

Furthermore, K_V_3.4-mediated I_A_ currents are dysfunctional in AD not only in neurons but also in glial cells [30,35,36,37]. In this respect, and in consideration that K_V_3.4 overexpression intervenes in the neuro-inflammation underlying AD development [30], BDS-I may assume a putative neuroprotective profile. Therefore, controlling the function of K_V_3.4 channels in AD brain with BDS-I might represent a novel therapeutic approach for slowing down the progression of the disease.

At higher concentrations than that used in the present manuscript, BDS-I is also an efficient modulator of Na_v_1.7 channel [46]. In fact, BDS-I slows down the inactivation Na^+^ channels, but slightly more than the specific toxins AsI, AsII, and AxI from *A. sulcata* and *A. xanthogrammica* [47,48]. Of note, BDS-I has only a small effect on tetrodotoxin (TTX)-sensitive Na^+^ channels [49] and no action on voltage-sensitive Na^+^ channels in cardiac cells or in skeletal muscle myotubes [39]. Therefore, putative downstream effects associated to its action on Na^+^ channels can be hypothesized, although restricted to the site of Na_v_1.7 localization.

The treatment of cultured astrocytes with exogenous Aβ oligomers induces a pathological remodeling of Ca^2+^ signaling [50] by affecting both neurotransmission machinery and ER Ca^2+^ handling [51]. In particular, under these conditions, changes in inositol 1,4,5-triphosphate receptor (IP_3_R) expression with consequent defects in store-operated calcium entry (SOCE) were recorded in astrocytes and in organotypic slices [24]. Of note, astrocytes isolated from 3xTg-AD mice displayed increased SOCE [52] and altered [Ca^2+^]_i_ oscillations [53]. Therefore, previous evidence showed the importance of astrocytic Ca^2+^ signaling in AD progression with particular respect to the intracellular component of Ca^2+^ machinery. In line with the burgeon literature on this issue, the present data show that BDS-I reduced ER Ca^2+^ overload in astrocytes induced by Aβ_1–42_ oligomers with the consequent prevention of the expression of ER stress markers, including active caspase 12 and GRP78/BiP. Indeed, calcium dysregulation, ER stress, and overexpression of unfolded protein response (UPR) elements like GRP78/BiP have been identified as common pathways in neurodegenerative diseases, including AD [24,54,55]. In particular, GRP78/BiP protein expression levels change differently according to the stage of AD: in chronic AD patients, GRP78/BiP expression is very low, while in the early AD stages, GRP78/BiP has been found to be overexpressed [43]. This highlights the putative role of the Ca^2+^-dependent ER chaperone as an early marker of the disease [43].

Of note, Ca^2+^ homeostasis and ER Ca^2+^ signaling in glia are under the control of other ionic players, including K^+^ channels as with Ca^2+^ signaling in a classical model of excitable cells [56]. Accordingly, deregulation of potassium homeostasis may underlie gliosis in AD. Mechanistically, the intermediate-conductance Ca^2+^-activated K^+^ channel K_Ca_3.1 may induce astrogliosis and microglia activation in the disease [57]. Furthermore, K_Ca_3.1, through the regulation of the membrane potential hyperpolarization, induces SOCE potentiation, ER Ca^2+^ overload, and the consequent ER stress in AD astrocytes [58]. Interestingly, gene deletion or pharmacological blockade of astrocytic K_Ca_3.1 reduce ER stress and prevent downstream neuronal loss in APP/PS1 mice [58], thus highlighting the importance of the astrocytic component in neuronal fate during AD progression.

Moreover, in the present study, the new role of astrocytic K_V_3.4 channel in the modulation of ER Ca^2+^ signaling has been highlighted in an in vitro model of AD.

In this respect, the mechanistic inquiry into AD pathogenesis and progression have recently switched from the neuron-centric doctrine to an astrocyte-centered theory [59]. Besides the evidence on the role of astrocytic K^+^ dysregulation during AD, with the present study, we could only partially answer to this enormous issue. In fact, many efforts should be made in the future to address this important question.

In conclusion, by investigating the relationship between K_V_3.4 channel and calcium signaling in astrocytes exposed to Aβ_1–42_ oligomers, the present study showed that: (i) dysregulation of K_V_3.4 channel induced an altered Ca^2+^ transient activity and (ii) the *Anemonia sulcata* toxin BDS-I prevented ER stress by reducing K_V_3.4 potassium channel hyperfunction and restoring spontaneous [Ca^2+^]_i_ transients, thus revealing a putative neuroprotective role for this marine toxin.

## 4. Materials and Methods

### 4.1. Drugs and Chemicals

Aβ peptides were synthesized by INBIOS (Naples, Italy). Rabbit polyclonal antibody against K_V_3.4 was from Alomone Labs (Jerusalem, Israel). Rabbit polyclonal antibody against caspase 12 was from Santa Cruz Biotechnology Inc. (Dallas, TX, USA). Rabbit polyclonal antibody against GRP78/BiP and rabbit monoclonal antibody against Aβ were from Cell Signaling Technology, Inc. (Danvers, MA, USA). Mouse monoclonal anti-β-actin, and rabbit polyclonal anti-GFAP antibodies, phalloidin-Atto Rho6G, Hoechst 33258, the reversed sequence Aβ_42–1_, trypsin, DNase, dimethyl sulfoxide (DMSO), 1,1,1,3,3,3-hexafluoro-2-propanol (HFIP), and (3[4,5-dimethylthiazol-2-y1]-2,5-diphenyltetrazolium bromide) (MTT) were from Sigma-Aldrich (Milan, Italy). Mouse monoclonal antibody against K_V_3.4 was from Anova (Walnut, CA, USA). Fura-2AM was from Calbiochem (Darmstadt, Germany). Enhanced chemiluminescent (ECL) reagents and nitrocellulose membranes were from GE Healthcare (Milan, Italy). Dulbecco’s modified Eagle’s medium (DMEM), fetal bovine serum, penicillin, streptomycin, and L-glutamine were from Gibco (Milan, Italy). Radioimmunoprecipitation assay (RIPA) buffer and protease inhibitor cocktail II were from Roche Diagnostic (Monza, Italy). Nitrocellulose membranes were from GE Healthcare.

### 4.2. Rat Primary Astrocytes

Primary cultures of rat astrocytes were obtained as previously described [60,61,62]. This protocol yields 98% of GFAP-positive cells. In brief, dissected cortices from 1- to 2-day-old rat pups were first dissociated enzymatically in a solution containing 0.125% trypsin and 1.5 mg/mL DNase and then, mechanically, in DMEM supplemented with 10% fetal bovine serum, 100 U/mL penicillin, 100 µg/mL streptomycin, and 2 mM L-glutamine. Cell pellets were plated on poly-L-lysine-coated plates. The medium was changed 24 h after plating and twice a week thereafter. For the mechanical dissociation, they were shaken vigorously to remove non-adherent cells and sub-cultured firstly at 1:3 dilution and then, once they reached confluency, at 1:4 dilution.

### 4.3. Solubilization of Aβ_1–42_ Peptide and Cellular Treatment

After synthesis, high-performance liquid chromatography and mass spectrometry showed a 95% purity for the yielded Aβ peptides. Lyophilized peptides were resuspended in HFIP at 1 mM, as previously described [63]. The clear solution was dried until complete elimination of the solvent and recovery of the dried powder. Immediately prior to use, the HFIP-treated aliquots were carefully and completely resuspended to a concentration of 5 mM in anhydrous dimethyl sulfoxide (Me_2_SO). Aβ_1–42_ and Aβ_42–1_oligomers were prepared by diluting the peptides at 5 mM concentration to a 100 µM solution in ice-cold cell culture medium (phenol red-free Ham’s F-12, 4 °C for 24 h). Then, the solution was centrifuged at 14,000 rpm at 4 °C for 10 min and the supernatant containing Aβ_1–42_ oligomers was stored at −20 °C [63,64]. Aβ_1–42_ preparation was tested with monoclonal anti-Aβ antibody, which recognizes an epitope within residues 17–42 of human Aβ (see Drugs and Chemicals Section). In particular, Western blotting showed a specific band at ~8 kDa, corresponding to Aβ_1–42_ dimers, and a smear ranging from ~8 to ~15 kDa, comprising lower molecular weight intermediates (trimers), at the highest concentration of Aβ_1–42_ preparation [63].

### 4.4. Western Blotting

Astrocytes were washed in phosphate-buffered saline (PBS) and collected by gentle scraping in ice-cold RIPA buffer containing, in mM: 50 Tris pH 7.4, 100 NaCl, 1 Ethylene glycol tetraacetic acid (EGTA), 1 phenylmethylsulfonyl fluoride (PMSF), 1 sodium orthovanadate, 1 NaF, 0.5% NP-40, and 0.2% Sodium dodecyl sulfate (SDS) supplemented with protease inhibitor cocktail II [65]. Then, protein samples were separated on 10% SDS-polyacrylamide gel and transferred onto nitrocellulose membranes. Membranes were incubated with rabbit polyclonal anti-K_V_3.4 (1:1000), rabbit polyclonal anti-caspase 12 (1:1000), rabbit polyclonal anti-GRP78/BiP (1:1000), and mouse monoclonal anti-β-actin (1:1000) antibodies. Immunoreactive bands were detected by chemiluminescence (GE Healthcare, Milan, Italy), and the software ImageJ (NIH, Bethesda, MD, USA) was used for densitometric analysis.

### 4.5. Immunohistochemistry

Immunostaining and confocal immunofluorescence procedures in cells were performed as previously described [56,57]. Cell cultures were fixed in 4% paraformaldehyde in PBS for 30 min and incubated in primary antisera, mouse monoclonal anti-K_V_3.4 (1:500), and rabbit polyclonal anti-GFAP (1:1000). Subsequently, they were incubated in a mixture of fluorescent-labeled secondary antibodies. Control experiments were performed as previously described [66,67]. Images were observed with a Zeiss LSM510 META/laser scanning confocal microscope (Gottingen, Germany). Single images were taken with an optical thickness of 0.7 µm and a resolution of 1024 × 1024. All images were obtained with a 40× objective with identical laser power settings.

### 4.6. Assessment of Nuclear and Cytoskeletal Morphology

Cytoskeleton morphology was studied by the staining of actin with rhodamine phalloidin at 1:50 dilution from stock solution of 100 µg/1 mL in PBS for 15 min at 37 °C [68]. Coverslips were mounted on glass slides and observed by fluorescence microscopy on a Nikon Eclipse E400 microscope (Nikon, Torrance, CA, USA). Digital images were taken with a CoolSnap camera (Media Cybernetics Inc., Silver Spring, MD, USA), stored on the hard-disk of a Pentium III computer, and analyzed with the Image-Pro Plus 4.5 software.

### 4.7. Electrophysiology

K^+^ currents were recorded from primary cortical astrocytes in control conditions, exposed to Aβ_1–42_ oligomers in the presence and in the absence of BDS-I with the patch-clamp technique in whole-cell configuration using a commercially available amplifier Axopatch 200B and Digidata 1322A interface (Molecular Devices, San Jose, CA, USA), as previously described [30,37]. In the same experimental conditions, current signals were acquired in gap-free modality using a Digidata 1322A interface using the protocol previously described [63]. Data were acquired using the pClamp software (version 9.0, Molecular Devices) and data analysis was performed using Clampfit software (version 9.0, Molecular Devices). Spontaneous action potential (AP) activity was measured in primary cortical astrocytes in control conditions, exposed to Aβ_1–42_ oligomers in the presence and in the absence of BDS-I using the protocol previously described [63,69,70]. Importantly, sustained high-quality whole-cell recordings could be maintained for >15 min with a stable membrane potential and AP waveform, confirming that the presence of spontaneous APs was not the result of declining cell health. Spontaneous AP amplitude and frequency were determined using our own computer program written in Java computer language. Briefly, for each primary cortical astrocyte, the software calculated the AP mean ± standard deviation (SD) during the baseline recording interval. This was used to define a cutoff identifying AP, which was set at mean AP ± 2SD. Subsequently, the software identified each value higher than this cut-off pointas AP. To quantify AP features in primary cortical astrocytes in control conditions, exposed to Aβ_1–42_ oligomers in the presence and in the absence of BDS-I, the following parameters were determined: the amplitude, defined as the difference between transient AP and mean basal and the frequency, defined as the number of peaks divided by the duration of observation. The pipette solution contained the following (in mM): 140 KCl, 2 MgCl_2_, 10 acido 4-2-idrossietil-1-piperazinil-etansolfonico (HEPES), 10 glucose, 10 EGTA, and 1 Mg-ATP adjusted at pH 7.4 with KOH. The extracellular solution contained the following (in mM): 150 NaCl, 5.4 KCl, 3 CaCl_2_, 1 MgCl_2_, 10 HEPES, adjusted pH 7.4 with NaOH. 50 nM tetrodotoxin (TTX) and 10 µM nimodipine were added to Ringer’s solution to abolish TTX-sensitive Na^+^, and L-type Ca^2+^ current. The blood-depressing substance-I (BDS-I; synthetized by Prof. P. Grieco, Department of Pharmacy, “Federico II” University of Naples, Naples, Italy) at the concentration of 50 nM was used to block K_V_3.4 currents [36,37,40].

Spontaneous action potential (AP) activity was measured in primary cortical astrocytes in control conditions, exposed to Aβ_1–42_ oligomers in the presence and in the absence of BDS-I using the protocol previously described [63,69,70]. Importantly, sustained high-quality whole-cell recordings could be maintained for > 15 min with a stable membrane potential and AP waveform, confirming that the presence of spontaneous APs was not the result of declining cell health. Spontaneous AP amplitude and frequency were determined using our own computer program written in Java computer language. Briefly, for each primary cortical astrocyte, the software calculated the AP mean ±standard deviation (SD) during the baseline recording interval. This was used to define a cutoff identifying AP, which was set at mean AP ± 2SD. Subsequently, the software identified each value higher than this cut-off pointas AP. To quantify AP features in primary cortical astrocytes in control conditions, exposed to Aβ_1–42_ oligomers in the presence and in the absence of BDS-I, the following parameters were determined: the amplitude, defined as the difference between transient AP and mean basal and the frequency, defined as the number of peaks divided by the duration of observation. The pipette solution contained the following (in mM): 140 KCl, 2 MgCl_2_, 10 acido 4-2-idrossietil-1-piperazinil-etansolfonico (HEPES), 10 glucose, 10 EGTA, and 1 Mg-ATP adjusted at pH 7.4 with KOH. The extracellular solution contained the following (in mM): 150 NaCl, 5.4 KCl, 3 CaCl_2_, 1 MgCl_2_, 10 HEPES, adjusted pH 7.4 with NaOH. 50 nM tetrodotoxin (TTX) and 10 µM nimodipine were added to Ringer’s solution to abolish TTX-sensitive Na^+^, and L-type Ca^2+^ current. The blood-depressing substance-I (BDS-I; synthetized by Prof. P. Grieco, Department of Pharmacy, “Federico II” University of Naples, Naples, Italy) at the concentration of 50 nM was used to block K_V_3.4 currents [36,37,40].

### 4.8. [Ca^2+^]_i_ Measurement

[Ca^2+^]_i_ was measured by single-cell computer-assisted video-imaging in astrocytes loaded with 10 µM Fura-2/AM [24,71,72]. [Ca^2+^]_i_ transients were identified using a software written in Java computer language, as previously reported [71]. Briefly, for each single astrocyte, the software calculated, during the time of recording, the [Ca^2+^]_i_ mean ± SD in order to define a cut-off point. Then, the software identified each value higher than this cut-off point as a single [Ca^2+^]_i_ transient. For each experiment, [Ca^2+^]_i_ transients were detected during the recordings and used to calculate the oscillation frequency that corresponds to the number of peaks divided by the duration of the recording (oscillation index).

### 4.9. Measurement of Reactive Oxygen Species

DCFH-DA, a cell membrane permeable fluorescein analogue, was used to detect ROS species production [36]. The rat primary astrocytes were pre-loaded with DCFH-DA (10 µM) for 30 min at 37 °C in PBS at the end of each pharmacological treatment. Cells were then washed with PBS, and the reaction was stopped by adding 2,6-di-tert-butyl-4-methylphenol (0.2% in ethanol) and EDTA (2 mM). The cells were then viewed with a Zeiss Axioscope 2FS plus fluorescence microscope (Gottingen, Germany) using excitation and emission wavelengths of 488 and 525 nm, respectively. Digital images were taken with a CoolSnap camera (Media Cybernetics Inc., Silver Spring, MD, USA), and analyzed with the Image-Pro Plus 4.5 software (Media Cybernetics Inc., Silver Spring, MD, USA). Image acquisition and processing were performed equally for all experimental conditions; for the quantification, background fluorescence was subtracted from the data.

### 4.10. Determination of Mitochondrial Activity

Mitochondrial dysfunction was evaluated with theMTT test [73,74]. In this test, the MTT dye is metabolized by viable mitochondria to a colored product and can be detected photometrically. Briefly, after the experimental procedures, rat primary astrocytes were washed with normal Krebs and incubated with 1 mL of MTT solution (0.5 mg/mL in PBS), as previously described [73,74]. After 1 h incubation at 37 °C, rat primary astrocytes were dissolved in 1 mL of DMSO, in which the rate of MTT reduction was measured using a spectrophotometer at a wavelength of 540 nm. Data are expressed as percentage of mitochondrial dysfunction versus sham-treated cultures.

### 4.11. LDH Release Assay

Cytotoxicity was detected using a LDH release assay kit (Jiancheng Bioengineering Institute of Nanjing, Jiangsu, China) according to the manufacturer’s protocol. Rat primary astrocytes were cultured in 96-well plates (1 × 10^4^ cells/well), and after treatment, supernatants were transferred to clean 96-well plates. Absorbance was analyzed at 450 nm using a Bio-Rad 680 Microplate Reader (Bio-Rad Laboratorie, Hercules, CA, USA) [75].

### 4.12. Statistical Analysis

Data are expressed as mean ± standard error of the mean (SEM). Statistical analysis was performed with unpaired *t*-test or one-way analysis of variance followed by Newman-Keuls test. Statistical significance was accepted at the 95% confidence level (*p* < 0.05).
